# Role of necroptosis in the pathogenesis of solid organ injury

**DOI:** 10.1038/cddis.2015.316

**Published:** 2015-11-19

**Authors:** H Zhao, T Jaffer, S Eguchi, Z Wang, A Linkermann, D Ma

**Affiliations:** 1 Anaesthetics, Pain Medicine and Intensive Care, Department of Surgery and Cancer, Faculty of Medicine, Imperial College London, Chelsea and Westminster Hospital, London, UK; 2 Division of Nephrology and Hypertension, Christian-Albrechts-University, Kiel, Germany

## Abstract

Necroptosis is a type of regulated cell death dependent on the activity of receptor-interacting serine/threonine-protein (RIP) kinases. However, unlike apoptosis, it is caspase independent. Increasing evidence has implicated necroptosis in the pathogenesis of disease, including ischemic injury, neurodegeneration, viral infection and many others. Key players of the necroptosis signalling pathway are now widely recognized as therapeutic targets. Necrostatins may be developed as potent inhibitors of necroptosis, targeting the activity of RIPK1. Necrostatin-1, the first generation of necrostatins, has been shown to confer potent protective effects in different animal models. This review will summarize novel insights into the involvement of necroptosis in specific injury of different organs, and the therapeutic platform that it provides for treatment.

## Facts

- Necroptosis is a type of regulated cell death dependent on the activity of receptor-interacting serine/threonine-protein (RIP) kinases
- Necroptosis induces inflammation through the release of Danger-associated molecular patterns (DAMPs), such as HMGB-1.
- Necroptosis is closely associated with acute injury in brain, heart, lung, kidney, liver, intestine and pancreas.

## Open Questions

- What is the exact molecular mechanism of necroptosis in solid organ injury?
- How is necroptosis related to diagnosis and prognosis of the solid organ injury?
- Which therapeutic strategy would be the most effective against necroptosis-associated solid organ injury in clinical settings?

Apoptosis and necrosis are two pathologically relevant types of cell death. Apoptosis is programmed cell death controlled tightly during development and in physiological cellular turnover; necrosis, which also occurs in trauma, was thought to occur predominantly in an uncontrolled manner.^1^ Necroptosis involves the loss of membrane integrity, release of damage-associated molecular pattern molecules (DAMPs) and is therefore closely associated with inflammatory response.^2^ It involves the activation of specific death mediators such as receptor-interacting protein (RIP) kinases and mixed-lineage kinase domain-like protein (MLKL).^3, 4^ Recent studies implicate that necroptosis is of central relevance in different disease states, such as myocardial infarction,^5^ stroke^6^ and organ graft ischemia–reperfusion injury (IRI).^7, 8, 9^ This review will summarize new insights into the molecular mechanisms of necroptosis in different pathological conditions and provide an overview of the currently available therapeutic approaches, which target necroptosis in organ injury.

## Molecular Mechanism of Necroptosis

**Figure 1:**
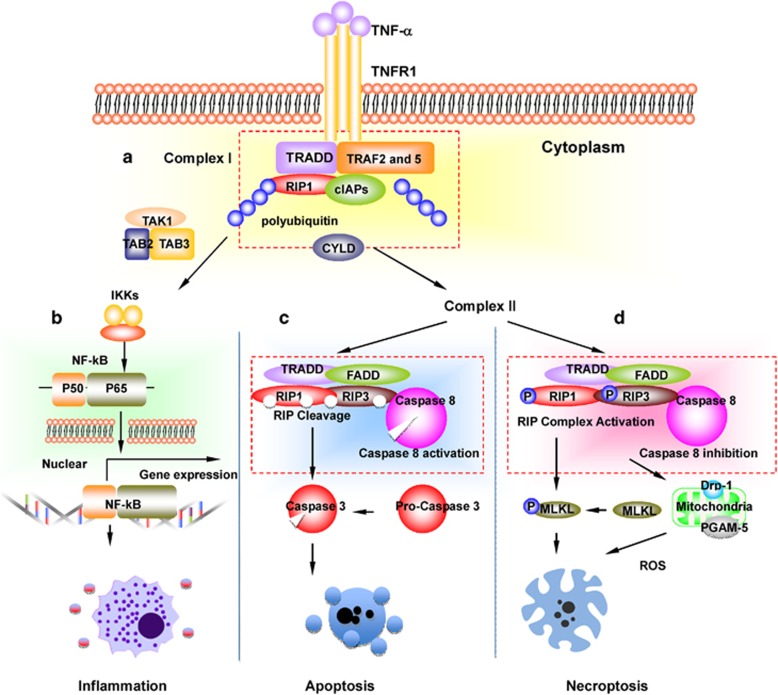
Signalling pathways after stimulation of the TNFR1. TNF-*α* is widely released during inflammatory conditions. (**a**) Upon binding to TNF receptor 1, TNF receptor 1 recruit TRADD, TRAF2 and 5, RIP-1, cIAPs and other molecules to form complex I. (**b**) Upon polyubiquitinated RIP-1, TNFR1-signalling activates NF-*k*B, leading to expression of proinflammatory cytokines. The deubiquitination of RIPK1 by CYLD leads to the formation of complex II. (**c**) Caspase-8 activation in complex II prevents necroptosis through preventing activation RIP, and suppresses the induction of necroptosis. (**d**) Inactivation of Caspase-8 in complex II leads to the phosphorylation and activation of RIPK1, RIPK3 and MLKL during the assembly of the necrosome. RIPK3 activates PGAM5 and Drp1 to induce reactive oxygen species (ROS) production in the mitochondria and mediate plasma membrane rupture. Abbreviation: TNF, tumor necrosis factor; TRADD, TNFRSF1A-associated via death domain; TRAF, TNF receptor associated factors; cIAPs, cellular inhibitor of apoptosis protein; CYLD, deubiquitinase cylindromatosis; MLKL, mediator mixed-lineage kinase domain like; RIP, receptor-interacting protein kinase; PGAM5, phosphoglycerate mutase 5; Drp1, dynamin-related protein 1

The molecular pathway of tumor necrosis factor alpha (TNF-*α*)-induced necroptosis is the mostly intensively investigated (Figure 1). TNF-*α*-induced necroptosis involves two members of the receptor-interacting protein (RIP) family of kinases – RIPK1 and RIPK3.^3, 10^ Tumor necrosis factor-*α* (TNF-*α*) can bind to one of two receptors, TNFR1 or TNFR2. TNFR activation can cause the activation of NF-*κ*B which leads to the induction of proinflammatory cytokines.^11^ However, the activation of TNFR1 may turn into a death signal, which recruits several proteins.^12^ Upon TNF stimulation, TNFR1 complex I is formed at the plasma membrane, containing cellular inhibitor of apoptosis protein 1 (cIAP-1) and transforming growth factor-*β*-activated kinase 1 (TAK1).^13^ The transition from TNFR1 complex I to the cytosolic death-inducing TNFR1 complex II requires the activity of cylindromatosis (CYLD), a deubiquitylating enzyme.^14^ More recent data^15, 16^ suggest that CYLD likely regulates activation of RIPK1 in Complex II, rather than transition from Complex I to Complex II. Within TNFR1 complex II, the apoptotic machinery FADD, c-FLIP and caspase-8 suppresses the induction of necroptosis. Caspase-8 inactivates RIPK1 and RIPK3 by proteolytic cleavage and the proapoptotic caspase activation is initiated.^12^ Mechanistic data indicate that CYLD is essential for necrosis and serve as a target for proteolysis by caspase-8.^17^ When caspase-8 is inactivated or absent, RIPK1 and RIPK3 are not cleaved and become phosphorylated.^1^ They then form a necrosome^18^ and the cell undergoes necroptosis.^12^ RIPK1 then recruits RIPK3 through RIP (RHIM) domain mediated-interactions. This RIPK1–RIPK3 interaction promotes the recruitment and phosphorylation of mixed-lineage kinase domain-like (MLKL) protein.^18, 19^ Phosphorylated MLKL forms tetramers and translocates onto the plasma membrane,^4, 19^ and in turn initiates Ca^2+^ influx.^19^ In addition, RIPK3 is reported to activate a number of different downstream signals such as phosphoglycerate mutase 5 and finally dynamin-related protein (Drp1) to induce reactive oxygen species (ROS) production in the mitochondria.^14^ The RIPK3-multimers are stabilized by chaperones that are essential for necroptosis.^20^

It is currently unclear which molecular switch mechanism controls the decision of whether apoptosis or necroptosis is executed, but first reports from TAK1-deficient cells suggest a critical importance of this kinase and its co-factors.^21^ Lamothe *et al.*^22^ reported that TNF-*α*-induced cell death was abrogated by the inhibition of caspases and knockdown of RIPK3 in TAK1-deficient mouse embryonic fibroblasts, indicating that TAK1 is an important modulator of both apoptosis and necroptosis. Saveljeva *et al.*^23^ demonstrated that repression of TNFR1, RIPK1 or MLKL shifted endoplasmic reticulum stress induced necroptosis to apoptosis. Necroptosis is caspase independent and is not inhibited by caspase inhibitors, such as zVAD fluoromethyl ketone.^7^ Necroptosis can be inhibited by Necrostatin-1 through suppression of RIPK1 activity.^24^ The difference between apoptosis, necrosis and necroptosis is summarized in Table 1.

RIPK1 has been shown to be involved in RIPK3/MLKL-dependent necroptotic cell death.^25, 26^ However, RIPK1 was found to drive NF-*κ*B-mediated cell survival and inflammation through activating downstream of either TNFR1 or TNFR2^27^ as well as caspase-8-dependent apoptotic cell death.^26^ Depletion of RIPK1 has been shown to lead to enhanced sensitization to TNF-induced apoptosis^26^ and pharmacological inhibition of RIPK1 activities is associated with suppression of necroptosis.^28^ Recent data clearly indicate that RIPK1 serves a critical kinase-independent scaffolding role and prevents inappropriate activation of both RIPK3-dependent necroptosis and caspase-8-dependent apoptosis.^25, 26, 27, 29, 30^

## Necroptosis and Inflammation

**Figure 2:**
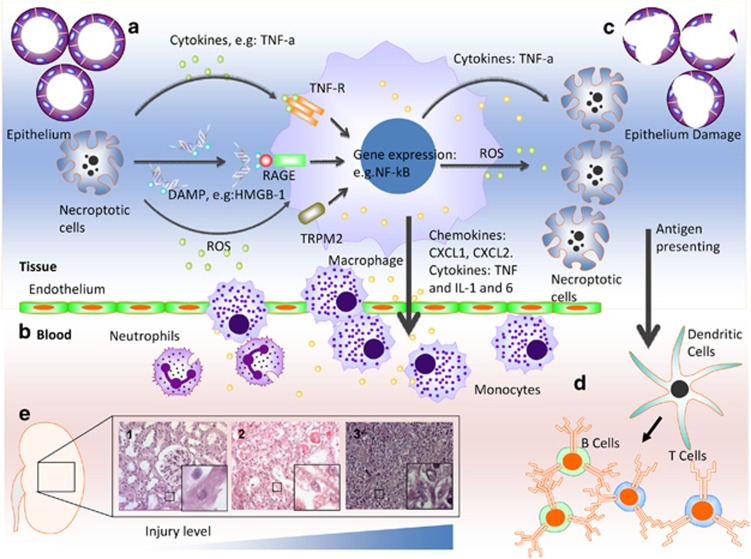
Necroptosis and inflammatory response. Necroptosis has been shown to commonly occur in the acute organ injury, especially in the organ transplantation. For example, during renal graft ischemia–reperfusion injury, (**a**) renal tubular epithelial cells undergoing necroptosis release DAMP molecules such as high-mobility group box 1 protein (HMGB-1), which is recognized by receptors, such as receptor for advanced glycation end products (RAGE) and Toll-like receptors 2, 4 and 9 (TLR-2, -4 and -9), reactive oxygen species (ROS) and inflammatory cytokines, such as TNF-*α*, leading to immune cell activation. (**b**) Inflammatory cells produce and release proinflammatory chemokine (e.g. CXCL1, CXCL2) and cytokines (e.g. IL-1 and -6) and promote tissue inflammation, infiltration of monocyte and neutrophils. (**c**) Infiltrating monocytes produce ROS and inflammatory cytokines such as TNF-*α* enhance the necroptosis in the epithelial cells. (**d**) Epithelial cells undergoing necroptosis could lead to the release and presentation of donor antigens by dendritic cells and activation of the acquired immune system, such as T cells and B cells. The immune rejection is then initiated. (**e**) Brown-Norway rat kidney was extracted and stored in UW solution for 24 h, and then transplanted into the Lewis recipient. Histology study demonstrated typical pathological progression possibly due to necroptosis. (1) Renal tubular cells. (2) Necrotic cells by ischemia–reperfusion injury 24 h after transplantation and (3) enhanced cellular infiltration during acute immune rejection 4 days after transplantation. The level of injury is gradually increased

Necroptosis is considered to be proinflammatory^31^ (Figure 2). Animal studies have revealed critical involvement for necroptosis in the pathogenesis of inflammatory diseases.^32^ Necroptosis is also demonstrated to lead to the release of damage-associated molecular patterns (DAMPs)^32^ (Table 2). DAMPs in the extracellular milieu trigger activation of the immune system and initiate inflammation. High-mobility group protein B1 (HMGB-1) is a typical DAMP molecule that initiates inflammation through toll-like receptors TLR-2 and TLR-4.^33^ Lau *et al.*^9^ demonstrated that RIPK1/RIPK3-mediated necroptosis regulates HMGB-1 release and HMGB-1 was reduced in RIPK3^−/−^ renal graft after injury. Further in-depth studies need to be carried out to identify DAMPs specific for necroptosis, which would provide better interpretation of the role of necroptosis in tissue inflammation and provide new therapeutic targets for inflammatory diseases.^34^

## Necroptosis in Organ Injury

**Figure 3:**
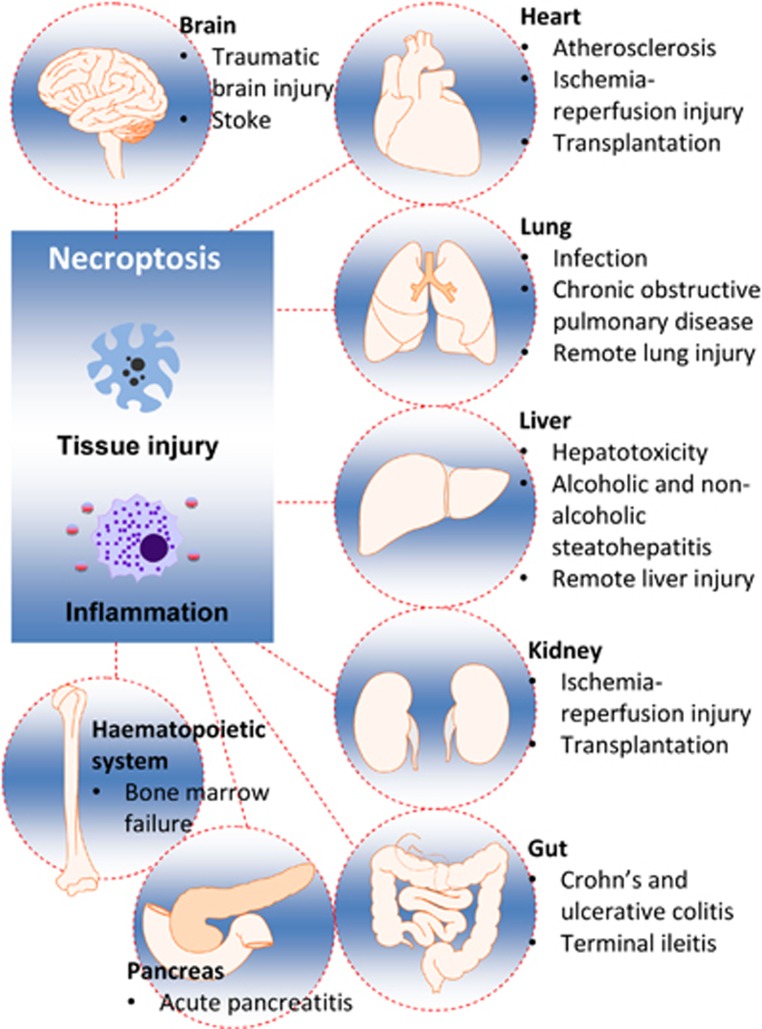
Necroptosis and organ injury. Necroptosis has been identified in various organs, including heart, lung, liver, kidney, gastrointestinal tract and central nervous system

Accumulating laboratory studies demonstrate that acute or chronic organ injury can lead to necroptosis.^35^ To date, necroptosis has been found to be present as a result of certain disease or injury in major organs of the body (Figure 3). Understanding of necroptosis in the pathophysiology of various diseases may offer unique opportunities for clinical management of such patients.

### Brain

#### Neonatal hypoxia–ischemia brain injury

Necroptosis has been recognized as an important mechanism of injury in neonatal hypoxia–ischemia (HI) brain injury.^36^ In the mouse model of neonatal HI,^37^ Necrostatin-1 blocked the interaction of RIPK1 and RIPK3 in the neurons. Cytokine gene and protein expression, such as interleukin IL-1*β*, IL-6 and TNF*α* was suppressed, and NF-*k*B activation was inhibited. This study showed that Necrostatin-1 could be protective against neonatal brain injury after HI through attenuation of necroptosis-associated neuro-inflammation.

#### Traumatic brain injury

Necroptosis has been shown to play a critical role in the pathogenesis of cell death during traumatic brain injury (TBI). Mice administered with Necrostatin-1 had reduced brain damage and improved motor and cognitive performance after TBI.^6^ Necrostatin-1 has been demonstrated to decrease brain neutrophil infiltration and suppress microglial activation.^6^ These data therefore suggest that Necrostatin-1 may offer a new therapeutic perspective for treatment of patients with TBI.

#### Ischemic stroke

The pathological relevance of necroptosis in an ischemic stroke setting was established in a study by Degterev *et al.*^38^ In a mouse model of transient focal cerebral ischemia, it was shown that necroptosis contributed to delayed ischemic brain injury. In addition, intracerebroventricular administration of Necrostatin-1 markedly decreased the infarct volume, indicating a therapeutic potential for stroke.

#### Hemorrhagic stroke

Although the role of necroptotic cell death after hemorrhagic stroke remains unexplored,^39^ the potential protective effect of Necrostatin-1 on such stroke has been investigated recently. In a mouse model of intracerebral hemorrhage,^40^ administration of Necrostatin-1 significantly reduced hematoma volume, neuronal cell death, reactive astrogliosis and neurovascular injury. The neurological outcomes were significantly improved after Necrostatin-1 treatment.

#### Amyotrophic lateral sclerosis

A study by Re *et al.*^41^ found that necroptosis is the key mechanism of neurodegeneration in both sporadic and familial types of amyotrophic lateral sclerosis (ALS). The sporadic and familial ALS astrocytes caused neuronal death by necroptosis in a RIPK1-dependent manner. Necrostatin-1 abrogated the mouse motor neuron loss in ALS.

#### Neurodegenerative disease

Necroptosis has been implicated in mediating neuronal excitotoxicity, which is associated with chronic neurodegeneration such as in Alzheimer's and Parkinson's diseases.^24^ Treatment with Necrostatin-1 protects against glutamate-induced oxytosis in hippocampal HT-22 cells.^42^ Furthermore, NMDA-induced excitotoxicity has been shown to be lowered in the rat cortical neurons through using Necrostatin-1.^43^ The molecule 24(*S*)-hydroxycholesterol (24*S*-OHC) was found to induce neuronal cell death via necroptosis in a neuroblastoma cell line.^44^ 24*S*-OHC is usually found as a cholesterol eliminator in brains, with increased levels in those patients with Alzheimer's disease.^45^ Treatment of cells with Necrostatin-1 significantly suppressed cell death induced by 24*S*-OHC. This indicated that high levels of 24*S*-OHC might lead to necroptosis of neurons and contribute to the development of neurodegenerative diseases.^46, 47^

### Lung

#### Lung infection

The cIAP may play a role in necroptosis within the lung during pulmonary infection.^48^ A study of necroptosis of macrophages demonstrated that with deficient cIAP-1, the mice had a greater *Chlamydophila pneumoniae* viral load, as well as a reduced number of macrophages.^48^ A deficiency in cIAP-1 would increase the risk of necroptosis due to increased formation of necrosomes. Flu infections caused necroptosis in the lung and bronchiole epithelial tissue,^49^ mediated by RIPK1 and RIPK3, and inhibiting either or both of these proteins in mice has shown to be effective against lung infection.^48^ Recently, the role of necroptosis in listeria infection has been pointed out in detail.^50^

#### Remote lung injury

Several reports have independently demonstrated that necroptotis is involved in remote lung injury. A recent study by Zang *et al.*^51^ examined rat lungs that were affected by acute kidney injury (AKI), and found that the lung cells with remote injury were undergoing both apoptosis and necroptosis. Furthermore, necroptosis was reported to be involved in remote lung injury after kidney graft IRI.^52^ In a rat renal allogeneic transplantation model, the expression of RIPK1 was enhanced in lung alveolar epithelial cells due to renal graft IRI, possibly due to enhanced local and systemic TNF-*α*.^52^ Blocking necroptosis through Necrostatin-1 conferred protection against remote lung injury after receiving ischemic renal allografts.^52^

#### Chronic obstructive pulmonary disease

Necroptosis was described to be involved in chronic obstructive pulmonary disease.^53^ In a study conducted by Mizumura *et al.*,^53^ cigarette smoke induced mitochondrial dysfunction in epithelial cells, and lung epithelial cells displayed increased expression of RIPK3. These findings implicated that necroptosis occurs in lung emphysematous changes in response to cigarette smoke exposure. The role of mitochondria in necroptosis has been discussed in more detail by Tait *et al.*,^54^ and they demonstrated that necroptosis is executed independently of mitochondrial permeability transition.

#### Transfusion-induced lung injury

Red blood cell transfusion had been shown to promote lung inflammation and induced necroptosis of lung endothelial cells.^55^ Necroptosis and the subsequent release of inflammatory mediators is a novel mechanism of injury following transfusion that may account for the increased risk of acute respiratory distress syndrome in such patients.^55^

### Kidney

#### Cisplatin-induced kidney injury

Necroptosis may be associated with the cisplatin toxicity-associated cell death^56^ and Necrostatin-1 has been shown to confer protection in cisplatin-treated human proximal tubular cells.^57^ In the mouse model of cisplatin-induced AKI, the deterioration of renal morphology was attenuated by Necrostatin-1.^20^ However, there are possible explanations, which include non-necroptotic functions of RIPK3 and off-target effects of Necrostatin-1 that might explain these findings which are not ruled out in detail. Renal tubular cells as such, when depleted from caspase-8 or FADD, are not sensitive to necroptosis in the cisplatin model.^58^

#### Renal ischemia–reperfusion injury

Recent work has further implicated the relevance of necroptosis in AKI, especially renal IRI.^59^ It has been demonstrated that Necrostatin-1 protects from renal IRI, a finding that suggests that RIPK1-dependent necroptosis is present and has functional relevance in the pathophysiological course of ischemic kidney injury.^28^ Importantly, this study indicated the absence of an apoptotic contribution to deterioration of acute ischemic kidney failure. To rule out potential off-target effects of Necrostatin-1 and non-necroptotic functions of RIPK3, the potential inhibition of RIPK1-mediated necroptosis by Necrostatin-1s, a stable and highly RIPK1 kinase specific compound, remains to be investigated. Preliminary studies do not indicate a protective effect of Necrostatin-1s in this very same model. The investigation of RIPK1-kd (kinase dead) knock-in mice is currently expected to clarify this issue.

#### Renal transplantation

RIPK3-mediated necroptosis was demonstrated to be present in renal transplantation and has a major impact on kidney transplant survival.^9^ TNF-*α* is expressed by infiltrating cells as well as kidney parenchymal cells during AKI, which enhanced RIPK3 expression. Enhanced survival was observed in RIPK3^−/−^ kidney graft recipients. Studies to investigate Necrostatin-1s and Necrostatin-1 in a transplant setting are urgently awaited, but are delayed due to the short half-life of these small molecules. Importantly, for the transplant situation in particular, the side effects upon immunosuppression, especially viral control, should be carefully looked at.

### Heart

#### Cardiac ischemia and infarction

It was reported^60^ that necrostatins inhibited myocardial cell death and reduced infarct size in the isolated perfused heart. In a study performed on guinea pig hearts, Koshinuma *et al.*^61^ looked at the effect of inhibition of necroptotic and apoptotic cell death on protection against ischemia injury. The combined treatment of necroptosis inhibitor Necrostatin-1 and apoptosis inhibitor zVAD led to reduced infarct size and a greater involvement of the RIPK1-mediated necroptosis pathway was demonstrated.^61^ In addition, RIPK3-deficient mice were strongly protected in a model of myocardial infarction.^5^ In a mouse model of cardiac IRI,^62^ Necrostatin-1 administration reduced infarct size, inhibited RIPK1/RIPK3 phosphorylation, and significantly reduced cell death. Therefore, Necrostatin-1 as a compound and RIPK3 as a therapeutic target currently attract tremendous interest in this pathway from pharmaceutical companies.

#### Cardiac transplantation

RIPK1 and RIPK3 were reported to contribute to cardiac allograft survival through necroptotic death.^8^ Necroptotic cell death and release of the danger molecule HMGB-1 were suppressed by Necrostatin-1 and by genetic deletion of RIPK3. RIPK3 deficiency in heart allografts led to less severe damage in the endothelium and myocytes, and prolonged graft survival after transplantation.

#### Atherosclerosis

RIPK3-dependent necroptosis in mouse models of atherosclerosis has been intensively investigated.^63^ It has been shown that RIPK3 has limited involvement in the early development of atherosclerosis, but has a promoting effect on advanced atherosclerosis. RIPK3 deficiency reduced inflammation and macrophage infiltration in advanced atherosclerotic lesions.

### Gastrointestinal tract

#### Crohn's and ulcerative colitis

RIPK1 was reported to regulate homeostasis and suppress inflammation in barrier tissues by inhibiting epithelial cell apoptosis and necroptosis.^64^ A study into children with inflammatory bowel disease showed that necroptosis might contribute to its progression by heightening intestinal inflammation.^65^ The study, by Pierdomenico *et al.*, found a statistically significant result in children with two main bowel diseases – namely Crohn's and ulcerative colitis. RIPK3 levels increased while caspase-8 decreased, which was a strong indicator for the presence of necroptosis. It is currently very suggestive to think of necroptosis as a cause of chronic inflammation in the gut, which might be dependent of RIPK3 and MLKL, potentially explaining the outstanding therapeutic success of infliximab, a drug that directly interferes with TNF-*α.*^66, 67^

#### Terminal ileitis

Gunther *et al.*^68^ demonstrate necroptosis in the terminal ileum of patients with Crohn's disease and suggest that regulating necroptosis in the intestinal epithelium is critical for the maintenance of intestinal immune homeostasis. Deletion of caspase-8 from intestinal epithelial cells led to spontaneous necroptosis, causing a Crohn's-like phenotype in mice.^68^ Paneth cells appear to be critically involved in this process. Mice with a conditional deletion of caspase-8 in the intestinal epithelium treated with Necrostatin-1 had a much higher survival rate than those that did not receive the treatment.

### Liver

#### Alcoholic steatohepatitis

The exact role of necroptosis in alcoholic steatohepatitis remains unclear. Roychowdhury *et al.*^69^ reported that ethanol feeding activates both apoptotic as well as non-apoptotic cell death pathways. Ethanol induced RIPK3 expression that was independent of presence or absence of caspase inhibitor. Chronic ethanol-fed mice showed increased RIPK3 expression. Moreover, the liver biopsies of alcoholic liver disease patients also showed increased RIPK3 expression, which indicates the execution of necroptosis in human hepatic pathologies.^69^

#### Non-alcoholic steatohepatitis

Gautheron *et al.*^70^ used the methionine- and choline-deficient diet-induced model of steatohepatitis as a model of non-alcoholic steatohepatitis. RIPK3 mediates liver injury, inflammation, induction of hepatic progenitor cells/activated cholangiocytes and liver fibrosis.^70^ RIPK3 thus represents a promising target for future therapeutic strategies in patients with chronic metabolic liver disease. It has been suggested that MLKL increases mitochondrial ROS production and contributes to necroptosis during hepatic injury.^71^

#### Hepatotoxicity

The role of Necrostatin-1 in protecting liver cell damage was recently investigated in the model of acetaminophen (APAP) induced hepatotoxicity.^72, 73^ APAP induced the phosphorylation of RIPK1 and hence necrosome formation, and Necrostatin-1 was effective in blocking necrosome formation during APAP-induced hepatotoxicity via inhibition of RIPK1.^72^

#### Liver infection

Recent studies highlight the critical role of necroptotsis in organ infection. Infection by *Listeria monocytogenes* induced the early necroptotic death of Kupffer cells, which caused monocyte recruitment and an antibacterial type 1 inflammatory response.^50^ Kupffer cell necroptosis triggered hepatocytes to release the alarmin interleukin-33 (IL-33) and enhanced basophil IL-4 production.^74^ Chronic hepatitis C virus (HCV) infection results in progressive liver fibrosis leading to cirrhosis and liver cancer. HCV promoted cell death in primary and immortalized hepatocytes, and this was inhibited by Necrostatin-1.^75^ These findings indicate that HCV-induced cell death occurs through necroptosis, and provides new insights into the mechanisms of HCV-induced liver injury.^75^

#### Remote liver injury

Necroptosis was shown to be present in remote hepatic injury associated with ischemic acute kidney injury (AKI).^76^ TNFR and RIPK3 showed significantly high expression levels in immunoblot analyses, and positive hepatocytes of RIPK3 immunohistochemical staining were also evident in livers of rats with ischemic AKI.

### Pancreas

#### Acute pancreatitis

RIPK3 deficiency was published by two initial reports in which the role of RIPK3 was highlighted^77, 78^ for partial protection from cerulein-induced pancreatitis (CIP), suggesting that necrotizing pancreatitis may be attenuated by pharmacological interference of this pathway.^78, 79^ However, other groups, who intended to reproduce this result, failed to do so and in fact observed stronger organ damage in the presence of Necrostatin-1.^79^ Recently, the same group also described that RIPK3-deficient mice are not protected from CIP.^58^ However, MLKL deficiency in a newly generated knockout mice was again shown to be responsible for the protection in the CIP model.^80^ In this regard, investigation of positivity for pMLKL in human pancreatitis sections would be very helpful to clarify the issue.

### Hematopoietic system

#### Bone marrow failure

A study by Roderick *et al.*^30^ revealed that hematopoietic RIPK1 deficiency triggered both apoptotic and necroptotic death that was partially prevented by RIPK3 deficiency, but the role of TAK1 was not investigated here. Therefore, the inhibitory function of RIPK1 on RIPK3-mediated necroptosis regulated hematopoiesis and prevented inflammation in this model. Interestingly, RIPK3^−/−^ bone-marrow-derived dendritic cells were highly defective in lipopolysaccharide (LPS)-induced expression of inflammatory cytokines.^81^ This dendritic cell -specific function of RIPK3 was critical for injury-induced inflammation and tissue repair.^81^ Rickard *et al.*^29^ demonstrated an essential physiological role for RIPK1 in immune homeostasis and emergency hematopoiesis. RIPK1 could inhibit RIPK3/MLKL necroptosis and RIPK3 and MLKL deficiency prevented RIPK1^−/−^ systemic inflammation. Specifically, bone marrow failure described by Roderick *et al.*^30^ and Rickard *et al.*^29^ reflects the loss of normal tissue homeostasis associated with the loss of RIPK1 protein and is different from the inappropriate activation of RIPK1 kinase activity. Similarly, the study by Moriwaki *et al.*^81^ demonstrated the kinase-independent role of RIPK3 in inflammation, which is again different from its kinase-dependent role in necroptosis.

## Necroptosis as a Therapeutic Target

### RIP-1 inhibitor

The central role of RIPK1 and RIPK3 in initiating necroptosis led to the assumption that inhibiting RIP is a good strategy against necroptosis.^82, 83^ Necrostatins in animal disease models have been proven to be very useful experimental probes.^84^ However, RIPK1 kinase activity is not only limited to necroptosis but is also involved in ERK^13^ and NF-*κ*B activation,^85^ therefore the biological effect after *in vivo* RIP inhibitor administration should be carefully interpreted.^86^ The kinase domain of RIPK1 appears to be of central importance in sepsis/LPS/TNF-mediated shock models.^25, 27^ A range of chemical or biological compounds have been proposed or investigated as potential inhibitors of RIPK1 in organ injury.

#### Necrostatin-1

Recently, the 5-(1H-indol-3-ylmethyl)-2-thiohydantoin 1, termed Necrostatin-1 (or Nec-1), was reported to inhibit necroptosis induced by TNF-*α*.^82, 87^ Necrostatin-1 has been considered an effective agent of necroptosis inhibition – possibly because the main cause of necroptosis is RIPK1 and RIPK3 forming the necrosome. However, this treatment so far has only been used in the preclinical stage, so further studies are needed for its development as a drug for clinical use. The precise function of the kinase-inhibitor Necrostatin-1 is well established that this molecule is an allosteric inhibitor of RIPK1, stabilizing a specific inactive conformation of the kinase domain.^82, 88^ It inhibits *in vitro* necroptosis and the *in vivo* effects seen with this compound are strong, and have been reported by several groups. RIPK1/3 activation is induced by homotypic RHIM domain interactions with upstream activators, such as TRIF.^89^ Loss of RIPK1 leads to the new parameters of direct RIPK3 activation by the same adaptors, which is clearly detrimental to the organism. However, this also indicates that the conclusion that necroptosis is RIPK1-independent has to be considered very carefully as regulation of RIPK3 in the presence of RIPK1 and its absence is not equivalent. Instead, it is clear that Necrostain-1 lacks activity in the absence of RIPK1, indicating its specific mode of action. Rather, these data indicate that RIPK1 has an important dual role in controlling cell death: both as an attenuator of RIPK3 activation under normal circumstances and as an inducer when necroptosis is activated under pathologic conditions. Thus, activation of necroptosis in RIPK1^−/−^ cells is not always a clear reflection of the lack of RIPK1 role under normal circumstances. In addition, RIPK1-kd knock-in mice are viable whereas whole-body RIPK1-ko are lethal at day 10.5 *in utero*.^26^ Conversely, unlike Necrostatin-1 derivatives such as Necrostatin-1s, Necrostain-1 is a reactive and to some extent non-specific inhibitor,^82, 90^ which may explain some discrepancies in the activities of the two molecules.

#### Necrostatin-1 derivatives

The new Necrostatin-1 analog, Necrostatin-1 stable (Necrostatin-1s) (7-Cl-O-Nec-1), was found to be 41 000-fold more selective for RIPK1 than for any other kinase.^91^ Moreover, a small group substitution at the seventh position of the indole of Necrostatin-1 and a change from thiohydantoin to hydantoin significantly enhanced its inhibitory activity.^82^ Necrostatin-1s was demonstrated to be very effective in reducing brain injuries^6, 38^

#### Other RIP-1 inhibitors

Harris *et al.*^92^ discovered some inhibitors for RIPK1 from three different molecule families. One of these families, furo[2,3-d]pyrimidines, has been shown to protect rats from hypothermia induced by TNF-*α*.^92^ Weng *et al.*^93^ demonstrated that RIPK1 inhibitor GSK′963, but not inactive enantiomer GSK′962, blocks *Yersinia pestis*-induced cell death and caspase-8 activity. Caspase-8 conditional KO macrophages are protected from *Y. pestis*-induced death in the presence of RIPK1 (GSK'963) or RIPK3 (GSK'872) kinase inhibitors.^93^

### RIPK3 inhibitor

The ability of RIPK3 deficiency to prevent disease has heightened interest in the therapeutic potential of small-molecule inhibitors that target RIPK3 kinase activity. Mandal *et al.*^94^ demonstrated that three selective small-molecule compounds (GSK′840, GSK′843 and GSK′872) to inhibit RIPK3-dependent necroptosis. These compounds interact with RIPK3 to activate caspase-8 (Casp8). RIPK3-inhibiting compounds blocked TNF-induced necroptosis in a concentration-dependent manner. In addition, RIPK3 inhibitors prevent death from a broader range of stimuli than RIPK1 inhibitors. Li *et al.*^95^ reported that RIPK3 activation following the induction of necroptosis required the activity of an HSP90 and a CDC37 cochaperone complex. Chemical inhibitors of HSP90 efficiently block necroptosis by preventing RIPK3 activation.

Potential cytotoxicity of RIPK3 inhibition compounds was recently observed and revealed by Mandal *et al.*^94^ Transmission electron microscopy confirmed apoptotic cell morphology associated with RIPK3i cytotoxicity, indicating that RIPK3 inhibition compounds could induce apoptotic cell death.

### Inhibitor of downstream signalling components

The downstream signalling cascade of RIPK1 may provide a significant array of novel candidate of therapeutic targets. This area certainly warrants further research.

#### MLKL inhibitor

The RIP downstream effector, MLKL, has been identified as another target of antinecroptosis therapy.^96^ A new compound named necrosulfonamide ((E)-*N*-(4-(*N*-(3-methoxypyrazin-2-yl) sulfamoyl) phenyl)-3-(5-nitrothiophene-2-yl) acrylamide), referred to as NSA, can effectively block TNF-induced necroptosis in human cells through interacting with MLKL.^4^ NSA could become a new drug for clinical applications in treating necroptosis related human disease.

## Conclusion

Based on effects seen in RIPK3-deficient mice, and on preclinical investigation of Necrostatin-1, a pathophysiological role of necroptosis in numerous diseases may be concluded.^97, 98^ Although the molecular mechanisms of necroptosis have not been fully explored, it is clearly possible that this process is involved in promoting organ injury. Thus, further understanding of the underlying mechanisms of necroptosis and its inhibition has important implications for organ protection and should have a significant impact on the development of therapeutic intervention of related human diseases.

## Figures and Tables

**Table 1:** Difference between apoptosis, necrosis and necroptosis

	**Apoptosis**	**Necrosis**	**Necroptosis**
Type of cell death	Controlled	Uncontrolled	Controlled
Trigger	Trauma, toxic stress, self-renew, aging, development.	Trauma, toxic stress, infection	Trauma, toxic stress, infection
Morphology	Extensive membrane blebbing, condensation and fragmentation of the nucleus^31^	Extensive organelle and cell swelling, loss of membrane integrity, release of extracellular contents^31^	Cytoplasmic swelling, rupture of the plasma membrane and spilling of the intracellular content^99^
Signalling pathway	Specific, intrinsic or extrinsic pathways	Unspecific	Specific, e.g TNFR1 pathway
Executioner	Caspase, (caspase-3, -6, -7, -8 and -9)		RIP kinase (RIPK1 and RIPK3)
Role of mitochondria	Release of cytochrome *c*, interaction with Bcl-2 protein family. Mitochondrial dysfunction	Mitochondrial dysfunction, collapse of mitochondrial membrane potential Failure of ATP production	Mitochondrial dysfunction, Production of ROS. AIF release
Complex formed	Apoptosome		Necroptosome
Inflammatory response	Anti- or proinflammatory response	Pro-inflammatory response	Pro-inflammatory response
DAMP release	Yes^100^	Yes	Yes
Inhibitor	Z-VAD fmk		Necrostatin-1
Human condition	Physiological or pathological condition	Pathological condition	Pathological condition

**Table 2:** A list of DAMP molecules possibly associated with necrotic or/and necroptotic cells

**Ligand**	**Receptor**
HMGB-1	RAGE, TLR-2, -4 and -9^101, 102^
Histone	TLR-2 and -4^103, 104^
ATP	P2X, P2Y^105^
HSP	CD24^106, 107^
DNA	TLR-9^108^
ECM components, e.g. fibronectin, fibrinogen, hyaluronan and biglycan	TLRs, CD44^107, 109^
IL-33	ST2L/IL1RAcP^34, 110^
Cyclophilin A	CD147^111, 112^

## Abbreviations

TNF: tumor necrosis factor
TRADD: TNFRSF1A-associated via death domain
TRAF: TNF receptor associated factors
cIAPs: cellular inhibitor of apoptosis protein
CYLD: deubiquitinase cylindromatosis
MLKL: mediator mixed-lineage kinase domain like
RIP: receptor-interacting protein kinase
Drp1: dynamin-related protein 1
TAK1: transforming growth factor-*β*-activated kinase 1
HMGB-1: high-mobility group protein B1
HSP: heat shock protein
DAMP: damage-associated molecular patterns
RAGE: advanced glycation end products
TLR: Toll-like receptors
AKI: acute kidney injury
